# Mixed-Methods Survey of Healthcare Workers’ Experiences of Personal Protective Equipment during the COVID-19 Pandemic in Aotearoa/New Zealand

**DOI:** 10.3390/ijerph19042474

**Published:** 2022-02-21

**Authors:** Cervantée E. K. Wild, Hailey Wells, Nicolene Coetzee, Cameron C. Grant, Trudy A. Sullivan, José G. B. Derraik, Yvonne C. Anderson

**Affiliations:** 1 Department of Paediatrics: Child and Youth Health, Faculty of Medical and Health Sciences, University of Auckland, 85 Park Road, Grafton, Auckland 1023, New Zealand; cervantee.wild@auckland.ac.nz (C.E.K.W.); hailey.wells@auckland.ac.nz (H.W.); nicolenec@outlook.com (N.C.); cc.grant@auckland.ac.nz (C.C.G.); j.derraik@auckland.ac.nz (J.G.B.D.); 2 Nuffield Department of Primary Care Health Sciences, University of Oxford, Radcliffe Primary Care Building, Radcliffe Observatory Quarter, Woodstock Road, Oxford OX2 6GG, UK; 3 General Paediatrics, Starship Children’s Hospital, 2 Park Road, Grafton, Auckland 1023, New Zealand; 4 Department of Preventive and Social Medicine, University of Otago, 18 Frederick Street, North Dunedin, Dunedin 9016, New Zealand; trudy.sullivan@otago.ac.nz

**Keywords:** personal protective equipment (PPE), healthcare worker, COVID-19

## Abstract

There have been widespread issues with the supply and distribution of personal protective equipment (PPE) globally throughout the COVID-19 pandemic, raising considerable public concern. We aimed to understand the experiences of healthcare workers using PPE during the first COVID-19 surge (February–June 2020) in Aotearoa/New Zealand (NZ). This study consisted of an online, voluntary, and anonymous survey, distributed nationwide via multimodal recruitment. Reported domains included PPE supply, sourcing and procurement, fit-testing and fit-checking, perceived protection, trust and confidence in the workplace, mental health, and the likelihood of remaining in the profession. Differences according to demographic variables (e.g., profession and workplace) were examined. We undertook a descriptive analysis of responses to open-text questions to provide explanation and context to the quantitative data. The survey was completed in October–November 2020 by 1411 healthcare workers. Reported PPE shortages were common (26.8%) among healthcare workers during surge one in NZ. This led to respondents personally saving both new (31.2%) and used (25.2%) PPE, purchasing their own PPE (28.2%), and engaging in extended wear practices. More respondents in the public system reported being told not to wear PPE by their organisation compared with respondents in the private sector. Relatively low numbers of respondents who were required to undertake aerosol-generating procedures reported being fit-tested annually (3.8%), a legal requirement in NZ. Healthcare workers in NZ reported a concerning level of unsafe PPE practices during surge one, as well as a high prevalence of reported mental health concerns. As NZ and other countries transition from COVID-19 elimination to suppression strategies, healthcare worker safety should be paramount, with clear communication regarding PPE use and supply being a key priority.

## 1. Introduction

The safety of healthcare workers has been a key area of focus during the COVID-19 pandemic [1], with healthcare workers being more likely to contract COVID-19 compared to the general public [2,3]. In addition, access to personal protective equipment (PPE), required to help prevent the transmission of COVID-19 to and between healthcare workers and patients, became a contentious issue worldwide as global supply chains were compromised [4,5]. NZ’s first COVID-19 surge was from 28 February 2020–8 June 2020, with 1504 cases and 22 deaths [6], when vaccination was not yet available. 

Concerns relating to PPE management occurred against a backdrop of reports identifying the need for health system reform in NZ. The 2020 Health and Disability System Review report highlighted the fragmented nature of the health system [7]. In terms of PPE management, this manifested in the 20 District Health Boards (DHBs—responsible for providing or funding the provision of health services in NZ within a given district) being made responsible for their own procurement and management of pandemic stock [8]. However, in June 2020, the NZ Office of the Auditor-General presented its report on the Ministry of Health’s management of PPE in response to COVID-19 [8]. The report concluded that there were gaps in the planning of PPE procurement and distribution, with insufficient national stock reserves (some of which had expired or was no longer fit for purpose), confusion around PPE use and extended use guidelines, and insufficient fit-testing of N95 respirator masks [8,9]. The PPE management process has since been centralised by the Ministry of Health [10]. 

Contemporary research appraising the experiences of healthcare workers elsewhere has identified limited PPE as a key issue in terms of occupational safety and increased self-reported anxiety during the pandemic response [11,12], highlighting the importance of the link between worker safety, mental health, and health workforce retention. This survey sought to describe the experiences of healthcare workers in NZ with regard to PPE access and usage during surge one of the COVID-19 pandemic in 2020. 

## 2. Methods

An anonymous web-based survey was developed by a multidisciplinary team of researchers including ‘frontline’ clinicians. Both closed (single response, multi-response, ranking, and Likert-scale questions) and open-text questions (extension, expansion, and general open questions) were used [13], as well as questions regarding demographic characteristics including age, gender, occupation, place of work, and ethnicity [14]. The intended respondents were healthcare workers who used medical PPE in their work. Multi-modal recruitment [15] was used to maximise its reach and the potential response, including distribution nationwide via mailing lists from professional and representative organisations; social and collegial networks; study advertisements on university and organizational websites; and word of mouth [15]. Some organisations agreed to distribute the survey via direct emails to their members or through periodic newsletters, or to post about the study on websites and social media channels. Of note, several organisations declined to participate in recruitment given the sensitivity of the subject matter. The survey was constructed using Qualtrics software (Qualtrics, Provo, UT, USA) and beta-tested before being distributed between 7 July and 30 November 2020. Electronic informed consent was obtained from all respondents, and those who did not provide their consent were unable to proceed to the survey.

Quantitative data were analysed using SPSS version 28 (IBM Corp., Armonk, NY, USA). Chi-square tests were used to compare the demographic characteristics with survey outcomes; specifically, comparisons were made between groups defined by age, sex, ethnic group, profession, and workplace (public or private). Statistical comparisons were two-tailed, and significance was set at *p* < 0.05.

The qualitative data collected expanded on specific questions (e.g., “Do you think frontline staff should have access to information regarding stock levels of PPE held by their organisation? Why/why not?”) (See Supplementary File S1) and were analysed with descriptive qualitative analysis [16] in nVivo v1.2 (QSR International Pty Ltd., Doncaster, Victoria, Australia). CEKW and NC collaborated to produce the coding categories and brief findings under question domains. Respondent quotations are provided in each section to provide context and are also presented in Supplementary Table S1; they have been ascribed to individual respondents ‘identified’ by their demographic characteristics. The names of any organisations were omitted from quotes to protect the anonymity of respondents.

Ethics approval was granted by the Auckland Health Research Ethics Committee (AH2640).

## 3. Results

### 3.1. Demographics

A total of 1411 survey responses were completed for analysis (257 incomplete responses, 1737 responses total received), and the demographic characteristics of respondents are reported in Table 1. Most respondents were female (81.6%), of NZ European ethnicity (73.9%), and lived in the North Island (76.1%) (Supplementary Table S1), with 74.1% aged ≥35 years. Respondents reported working in allied health (34.4%), nursing (33.2%), medical (19.1%), dental (6.1%), and other areas of health (7.2%). Seventy-three respondents (5.2%) reported having contracted COVID-19, while 159 (11.3%) reported knowing a relative/friend who had contracted COVID-19. Of respondents, 35.3% reported being required to undertake aerosol-generating procedures (AGPs) in their work.

### 3.2. PPE Supply

Table 2 summarises issues relating to reported PPE supply during surge one in respondents’ areas of work. Thirty-eight percent were often or always aware of shortages or very low levels of PPE in their workplace; however, 68.3% reported receiving adequate PPE from their organisation to do their job at all times. 

Nearly one in four respondents reported being told not to wear PPE by their organisation for reasons relating to stock levels (24.4%) or for other reasons (20.8%). A larger proportion of respondents working in the public versus the private healthcare sector recalled being told not to wear PPE by their organisation for reasons related to stock levels (public vs. private, 28.0% vs. 18.2%; *p* < 0.001) and for other reasons (public vs. private, 25.4% vs. 13.4%; *p* < 0.001). The latter included wearing PPE would create panic (“It would scare the public if we wore masks.”—*Consultant doctor, female, 35–44 years, NZ European*); it was unnecessary or that staff were not at risk; and the cost of PPE was too high (see Supplementary Table S1).

Over half of respondents (61.2%) reported confusion around adequate PPE supply, with 80.6% agreeing that frontline staff should have access to information regarding stock levels at their organisations. Reasons for and against providing staff with such information are reported in Supplementary Table S1. Reasons in favour of providing staff with access to stock level information included process reasons (it would help understand decision making and better prioritise resources), welfare reasons (it would reassure healthcare workers, promote trust, and make staff feel valued), and rights-based arguments (staff have a right to the information and could choose not to work if they felt unprotected):

*“Communication and transparency reduces [sic] anxiety and misinformation spreading.”*

—Dentist, female, 65+ years, NZ European

*“Team effort-everyone then feels empowered by their knowledge and understand the situation better.”*

—Nurse, female, 45–54 years, Māori

Nearly 40% of respondents reported their organisation could improve their communication regarding PPE use and supply.

### 3.3. Sourcing and Procurement

Survey items related to the sourcing and procurement of PPE are presented in Table 3. Respondents reported saving both new (31.8%) and used (25.2%) PPE in the event of their organisation running out of PPE. Of the 28.3% of respondents who reported personally buying PPE for themselves or their department, 60.5% reported being allowed to wear them at their workplace. Those who reported not being allowed to do so were told it would create panic, was unnecessary, was not approved by the workplace, or that they would pose a contamination risk (Supplementary Table S1):

*“Management stated it was ‘unnecessary and undermining confidence in staff from families’.”*

—Nurse, female, 65–44 years, NZ European

*“It was not provided via supply lines that the organisation has approved and could be confident in.”*

—Nurse practitioner, male, 35–44 years, NZ European

Respondent mask-wearing behaviour is shown in Table 4. Over half (*n* = 788, 55.8%) reported wearing filtering facepiece respirators (FFRs) (N95-type masks) and 1296 (91.8%) reported wearing surgical masks during at least one work shift during surge one. Among respondents who reported using FFRs for uninterrupted periods (*n* = 498), 15.9% of respondents reported having worn these for more than 4 h, with 3.2% reporting 8–12 h of continuous FFR usage (Table 4).

### 3.4. Fit Testing and Fit Checking

Among the respondents who reported being required to undertake AGPs, (35.3%; *n* = 498), 61.6% reported being fit-tested for an FFR in 2020, with 3.8% reporting annual fit-tests prior to the pandemic (Table 5). Gender was not associated with having failed a fit-test (10.2% females vs. 9.0% males; *p* = 0.59). Over half of respondents (54%) who had failed a fit-test were not informed how the organisation would ensure access to a well-fitting FFR. Consequently, 79% of these respondents experienced worry or undue stress related to a lack of clarity over protection at work.

### 3.5. Perceived Protection, Trust, and Confidence in Workplace

On a scale of 0 (no trust) to 10 (complete trust), respondents’ median level of trust in their organisations to provide enough PPE for all workers who required it during surge one was 7 (IQR = 4–9). Sixty-two percent of respondents reported feeling somewhat or very protected at work during surge one, while 27.9% felt somewhat or very unprotected. Nearly half (49.0%) of respondents reported confidence in their workplace to provide PPE in case of further surges of COVID-19. Confidence in the workplace was not associated with profession or place of work. Reported reasons for a lack of confidence or uncertainty are shown in Supplementary Table S1. Historical access issues, known high burn rate (average consumption rate), poor quality PPE available, previous failed fit-tests, lack of transparency and trust in the organisation, and a feeling of being dispensable were reported:

*“Because we are already struggling for the appropriate PPE that we need on a daily basis.”*

—Dental hygienist, female, 21–34 years, NZ European

*“I no longer trust them. We still don’t have consistent N95 supply.”*

—Consultant doctor, female, 35–44 years, NZ European

### 3.6. Mental Health

Table 6 presents data describing the prevalence of self-reported mental health concerns during surge one. Of those reporting at least one mental health concern, 51.1% reported that these were not present prior to the pandemic. The proportion of females reporting at least one mental health concern was greater than that of males (64.6% vs. 56.1%, respectively; *p* = 0.011). In addition, 31.0% reported experiencing increased anxiety over the availability of PPE at work; this rate was slightly higher in respondents working in the private than in the public sector (35.8% vs. 30.4%, respectively; *p* = 0.036), and higher in those working in dental vs. non-dental fields (47.1% vs. 31.4%; *p* = 0.003). 

### 3.7. Likelihood of Remaining in Health Profession

When asked about the effects of the COVID-19 pandemic on the likelihood of continuing to work in healthcare, 3 in 4 respondents (74.3%) reported that the pandemic made no difference. In contrast, 15.9% (*n* = 225) reported they were somewhat or much less likely to remain in healthcare, while 9.7% (*n* = 147) reported being somewhat or much more likely to do so. Respondents who indicated that they would remain in the health profession cited job security and satisfaction, low perceived risk at work, that pandemics were ‘part of the job’, or that they had no alternative options (Supplementary Table S1).

*“Healthcare is what I do. This is just another aspect of my work. Somewhat challenging times but maybe that’s what makes it more rewarding.”*

—Nurse, female, 55–64 years, NZ European

Respondents intending to leave reported that the profession was too risky, having high levels of stress, feeling burnout, undervalued, and/or under-resourced; some of those working in private practice also mentioned that the profession was financially untenable.

*“I have underlying health conditions and am worried I would die if I contracted Covid.”*

—Pharmacist, female, 45–54 years, NZ European

*“Lack of government support for role [of] general practice. We stayed open at huge personal and financial cost.”*

—General practitioner, female, 55–64 years, NZ European

## 4. Discussion

This study shows that PPE shortages were commonly reported among healthcare workers in NZ during surge one of the COVID-19 pandemic. PPE was reused with no clear procedures in place. In addition, healthcare workers feared running out of PPE, saving both new and used PPE and purchasing their own. Healthcare workers were also uncertain that their organisations would be able to keep up with demand. 

Communication regarding PPE stock and use could have been improved. A qualitative study of frontline healthcare workers in the United Kingdom identified that a key concern was running out of PPE, and that frequently changing clinical guidelines were confusing and led to distrust in the messaging [17]. Those observations were reflected in our study, as while 68.4% of respondents reported having adequate PPE to perform their job, 60.6% also reported being confused about supply. In addition, 80.3% of respondents agreed that frontline staff should have access to information regarding PPE stock levels. Open-text responses indicated this information would help staff better understand decision making and promote trust within their organisation, suggesting that transparency and clear communication in the workplace are vital to mitigate confusion and distrust. This reflects what was previously found during the SARS outbreak in 2002–2004 [18]. Communication is critical to trustworthy leadership and healthcare worker welfare in a pandemic situation.

Notably, respondents reported several legal and ethical concerns regarding workplace occupational health and safety. Of the 498 respondents who reported having to undertake AGPs, only 3.8% reported undergoing annual fit-tests prior to the COVID-19 pandemic. Annual fit-testing is a legal requirement for workers undertaking AGPs in NZ [19], as in other countries [20]. Given the challenges created by stock levels, it is important that individuals are fit-tested to FFR models available in their organisation, fit-tested again if models change, and are adequately informed of their options should they not pass a fit test for an FFR at work. While these requirements do pose logistical issues for health organisations with large numbers of staff, they are nonetheless fundamental aspects of occupational health and safety in certain healthcare environments.

The fact that both the extended uninterrupted wear of >4 h and intermittent wear of FFRs were prevalent among survey respondents is also important to note, given this is not recommended practice [10]. Almost one quarter (23.5%) of respondents reported being told by their organisation not to wear PPE due to stock levels, and 21% reported being told not to wear PPE for other reasons. These proportions were higher for those working in the public sector than those working in the private sector. It is also noteworthy that multiple respondents reported being told not to wear PPE because it was unnecessary or would create panic among the general public. It is acknowledged that this was a time of rapidly mobilising a workforce to manage COVID-19-infected patients, producing guidelines on when to use PPE, and balancing appropriate use and supply. This study did not examine whether these instances occurred when PPE use was indicated or not; however, it does highlight the importance of clear communication in such circumstances.

The need to prevent and manage occupational stress and burnout in a pandemic scenario has previously been documented [21,22,23,24]. The self-reported mental health status of healthcare workers in our survey is concerning. Sixty-two percent experienced at least one mental health concern, with 54.1% of healthcare workers reporting that these issues were not present prior to the COVID-19 pandemic. Our findings reflect observations elsewhere [25,26]; a survey conducted in April-May 2020 in Melbourne (Australia) showed marked levels of depression, anxiety, and post-traumatic stress disorder among healthcare workers, despite the relatively low levels of COVID-19 exposure [26]. In NZ, a survey of healthcare workers’ mental health during the COVID-19 pandemic found that 71% were at a greater risk of moderate levels of anxiety than non-essential workers [27]. 

This study has important implications for future workforce considerations, as 15% of respondents indicated they were less likely to remain in the health profession as a result of the COVID-19 pandemic. Given NZ is currently experiencing considerable healthcare workforce shortages [7], understanding whether this figure has changed as the COVID-19 pandemic continues warrants further examination. 

This survey is limited by the use of self-reported outcomes, which may not be as robust as diagnosed mental illness prevalence data, for example. The survey was undertaken several months after surge one, so it may be affected by recall bias. There was reasonably low participation from male and Māori respondents, although this was in line with 2015 workforce proportion estimates [28] (no more recent data available). This study only reports on the experiences of healthcare workers; future research should also capture the accounts of other ‘frontline’ workers, such as police, fire service, and border-facing employees. The strengths of this survey were the large number of respondents from across NZ, and the use of both quantitative and qualitative analysis methods.

## 5. Conclusions

In conclusion, healthcare workers in NZ reported a considerable number of non-recommended practices regarding PPE in this survey, as well as a high prevalence of mental health concerns. PPE was reused and saved in the case of running out. In addition, responses suggested evidence of PPE mismanagement on the part of organisations at a time when stress was heightened. As NZ and other countries transition from elimination to suppression strategies, healthcare worker safety and wellbeing are critical in the event of future surges, with clear communication regarding PPE use and supply being a key priority.

## Figures and Tables

**Table 1:** Demographic characteristics of survey respondents.

*n* ^a^		1411
Gender	Female	1140 (81.6%)
Ethnicity ^b^	NZ European	995 (73.9%)
	Asian	190 (14.1%)
	Māori	102 (7.6%)
	Other ^c^	60 (4.5%)
Age	<35 years	366 (25.9%)
	35–44 years	299 (21.2%)
	45–54 years	346 (24.5%)
	≥55 years	400 (28.3%)
Profession	Allied health	486 (34.4%)
	Nursing	468 (33.2%)
	Medical	269 (19.1%)
	Dental	86 (6.1%)
	Other health	102 (7.2%)

^a^ Some respondents did not answer all questions in the survey, so the *n* for individual parameters is lower than the total number of responses received. ^b^ Prioritised ethnicity output categories [14]. ^c^ ‘Other’ ethnicity includes Pacific Peoples.

**Table 2:** Personal Protective Equipment (PPE) supply during COVID-19 surge one by respondents’ areas of work.

*n*	1411 ^a^
Awareness of shortages or very low levels of PPE	
Always	208 (15.4%)
Often	304 (22.6%)
Sometimes	458 (34.0%)
Rarely	213 (15.8%)
Never	164 (12.2%)
Received adequate PPE from organisation to do their job at all times	
Yes	920 (68.3%)
No	361 (26.8%)
Don’t know	66 (4.9%)
PPE items that were missing or in low supply	
FFRs	669 (47.4%)
Face shields	481 (34.1%)
Surgical masks	468 (33.2%)
Gowns	389 (27.6%)
Eyewear	353 (25.0%)
Other ^b^	111 (7.9%)
Confusion regarding adequate supply of PPE	
Yes	825 (61.2%)
No	365 (27.1%)
Don’t know	157 (11.7%)
Told by organisation not to wear PPE due to stock levels	
Yes	328 (24.4%)
No	950 (70.5%)
Don’t know	69 (5.1%)
Told by organisation not to wear PPE for other reasons	
Yes	208 (20.8%)
No	974 (72.3%)
Don’t know	93 (6.9%)
Frontline staff should have access to information regarding stock levels of PPE held by their organisation	
Yes	1086 (80.6%)
No	106 (7.9%)
Don’t know	155 (11.5%)
Organisation could improve communication regarding PPE use and supply	
Yes	503 (37.6%)
No	368 (27.5%)
Don’t know	467 (34.9%)

FFR, filtering facepiece respirators (N95-type ‘masks’); PPE, personal protective equipment. ^a^ Some respondents did not answer all questions in the survey, so the *n* for individual parameters is lower than the total number of responses received. ^b^ “Other” included: missing gloves, sanitisers, head coverings, scrubs and washable aprons, foot coverings, fit or quality issue, did not know what was missing, or in low supply.

**Table 3:** Sourcing and procurement of personal protective equipment (PPE) during COVID-19 surge one.

*n*	1411
Personally saved items of used PPE for use in the event of organisation running out	
Yes	340 (25.2%)
No	977 (72.5%)
Prefer not to answer	30 (2.2%)
Personally saved items of new PPE for use in the event of organisation running out	
Yes	420 (31.2%)
No	901 (66.9%)
Prefer not to answer	26 (1.9%)
Purchased PPE directly for themselves or department	380 (28.2%)
Items purchased: ^a^	
Surgical masks	252 (66.3%)
FFRs	157 (41.3%)
Eyewear	139 (36.6%)
Face shields	136 (36.6%)
Gowns	84 (22.0%)
Other ^b^	102 (26.8%)
*If purchased*: Allowed to wear purchased PPE at work	
Yes	233 (61.3%)
No	22 (5.8%)
Don’t know	29 (7.6%)
I was not in a situation where this was necessary	96 (25.3%)
Participant or department received PPE donated by charity(ies), local firm(s), or individual donor(s)	
Yes	152 (11.3%)
No	673 (50.0%)
Don’t know	522 (38.8%)

FFR, filtering facepiece respirators (N95-type ‘masks’). ^a^ Multiple responses possible. ^b^ “Other” included: gloves, footwear, other masks, headwear, garments, and sanitizers.

**Table 4:** Longest reported amount of time using N95-type filtering facepiece respirators (FFRs) and surgical masks during COVID-19 surge one.

Extended Wear Type	Intermittent	Uninterrupted
Time wearing FFRs (*n* = 788)		
*n*	290	498
<4 h	166 (57.2%)	419 (84.0%)
≥4 h	124 (42.8%)	79 (16.0%)
Time wearing surgical mask (*n* = 1296)		
*n*	666	630
<4 h	234 (35.1%)	429 (68.1%)
≥4 h	432 (64.9%)	201 (31.9%)

**Table 5:** Filtering facepiece respirator (FFRs) testing, knowledge and skills.

*n*	498
Fit tested for an FFR this year [2020]	307 (61.6%)
Fit tested annually prior to the COVID-19 pandemic	19 (3.8%)
Knowledge of difference between a fit test and a fit check of an FFR ^a^	223 (44.8%)
Taught how to undertake a fit check of an FFR in current place of employment	281 (56.4%)
Ever failed fit test for an FFR at current place of employment	113 (22.7%)
Have been informed how organisation will ensure access to a well-fitting FFR ^b^	52 (46.0%)
If answered “no” or “don’t know”: Causes worry or undue stress	48 (78.7%)

FFR, filtering facepiece respirators (N95-type ‘masks’). ^a^ Prior to taking this survey. ^b^ *n* = 58 answered “no”; *n* = 3 answered “don’t know”.

**Table 6:** Prevalence of self-reported mental health issues among survey respondents during COVID-19 surge one.

*n* ^a^	1395
Prevalence of at least one self-reported mental health symptom during COVID-19 surge one ^b^	877 (62.9%)
Fatigue	577 (41.4%)
Stress	536 (38.4%)
Burnout	394 (28.2%)
Anxiety	369 (26.5%)
Emotional distress	221 (15.8%)
Depression	176 (12.6%)
Other ^c^	27 (2.0%)
Symptom(s) present prior to the pandemic	
Yes	358 (40.4%)
No	463 (52.3%)
Don’t know	65 (7.3%)
Increased anxiety over availability of PPE at work ^d^	
Yes	455 (32.3%)
No	910 (64.6%)
Don’t know	43 (3.1%)

PPE, personal protective equipment. ^a^ *n* = 16 chose not to answer this question. ^b^ Multiple responses possible. ^c^ “Other” included: frustration, disappointment, boredom, sleep issues, bipolar disorder, post-traumatic stress disorder, eating disorder, and other not further defined. ^d^ *n* = 3 chose not to answer this question.

## Data Availability

Data are not available.

## Supplementary Materials

The following supporting information can be downloaded at: https://www.mdpi.com/article/10.3390/ijerph19042474/s1, File S1: Online Questionnaire Open Questions; Table S1: Question domain, Reported findings and Supporting data example

## References

[1] Shaw A., Flott K., Fontana G., Durkin M., Darzi A. (2020). No patient safety without health worker safety. Lancet.

[2] Nguyen L.H., Drew D.A., Graham M.S., Joshi A.D., Guo C.-G., Ma W., Mehta R.S., Warner E.T., Sikavi D.R., Lo C.-H. (2020). Risk of COVID-19 among front-line health-care workers and the general community: A prospective cohort study. Lancet Public Health.

[3] Mutambudzi M., Niedzwiedz C., Macdonald E.B., Leyland A., Mair F., Anderson J., Celis-Morales C., Cleland J., Forbes J., Gill J. (2021). Occupation and risk of severe COVID-19: Prospective cohort study of 120 075 UK Biobank participants. Occup. Environ. Med..

[4] Cohen J., Rodgers Y.V.M. (2020). Contributing factors to personal protective equipment shortages during the COVID-19 pandemic. Prev. Med..

[5] Livingston E., Desai A., Berkwits M. (2020). Sourcing personal protective equipment during the COVID-19 pandemic. JAMA.

[6] Ministry of Health COVID-19—Current Cases. https://web.archive.org/web/20200608205411/https://www.health.govt.nz/our-work/diseases-and-conditions/covid-19-novel-coronavirus/covid-19-current-situation/covid-19-current-cases.

[7] Health and Disability System Review (2020). Health and Disability System Review—Final Report. Pūrongo Whakamutunga.

[8] Controller and Auditor-General (2020). Ministry of Health: Management of Personal Protective Equipment in Response to COVID-19.

[9] Jones N. (2021). Exclusive: Auckland Hospital Nurses Told to Wear Unfitted N95 Masks Because of Backlog.

[10] New Zealand Ministry of Health COVID-19: Personal Protective Equipment Central Supply. https://www.health.govt.nz/our-work/diseases-and-conditions/covid-19-novel-coronavirus/covid-19-information-specific-audiences/covid-19-personal-protective-equipment-central-supply.

[11] Vindrola-Padros C., Andrews L., Dowrick A., Djellouli N., Fillmore H., Bautista Gonzalez E., Javadi D., Lewis-Jackson S., Manby L., Mitchinson L. (2020). Perceptions and experiences of healthcare workers during the COVID-19 pandemic in the UK. BMJ Open.

[12] Willis K., Ezer P., Lewis S., Bismark M., Smallwood N. (2021). “Covid Just Amplified the Cracks of the System”: Working as a Frontline Health Worker during the COVID-19 Pandemic. Int. J. Environ. Res. Public Health.

[13] O’Cathain A., Thomas K.J. (2004). “Any other comments?” Open questions on questionnaires—A bane or a bonus to research?. BMC Med. Res. Methodol..

[14] Ministry of Health (2017). HISO 10001:2017 Ethnicity Data Protocols.

[15] McRobert C.J., Hill J.C., Smale T., Hay E.M., van der Windt D.A. (2018). A multi-modal recruitment strategy using social media and internet-mediated methods to recruit a multidisciplinary, international sample of clinicians to an online research study. PLoS ONE.

[16] Bradshaw C., Atkinson S., Doody O. (2017). Employing a Qualitative Description Approach in Health Care Research. Glob. Qual. Nurs. Res..

[17] Hoernke K., Djellouli N., Andrews L., Lewis-Jackson S., Manby L., Martin S., Vanderslott S., Vindrola-Padros C. (2021). Frontline healthcare workers’ experiences with personal protective equipment during the COVID-19 pandemic in the UK: A rapid qualitative appraisal. BMJ Open.

[18] Nicolle L. (2003). SARS safety and science. Can. J. Anaesth..

[19] (2009). Australian/New Zealand Standard: Selection, Use and Maintenance of Personal Protective Equipment.

[20] National Institute of Occupational Health and Safety (2013). Respirator Awareness: Your Health May Depend on It.

[21] Sriharan A., Ratnapalan S., Tricco A.C., Lupea D. (2021). Women in healthcare experiencing occupational stress and burnout during COVID-19: A rapid review. BMJ Open.

[22] Roycroft M., Wilkes D., Fleming S., Pattani S., Olsson-Brown A. (2020). Preventing psychological injury during the COVID-19 pandemic. BMJ.

[23] Trumello C., Bramanti S.M., Ballarotto G., Candelori C., Cerniglia L., Cimino S., Crudele M., Lombardi L., Pignataro S., Viceconti M.L. (2020). Psychological adjustment of healthcare workers in Italy during the COVID-19 pandemic: Differences in stress, anxiety, depression, burnout, secondary trauma, and compassion satisfaction between frontline and non-frontline professionals. Int. J. Environ. Res. Public Health.

[24] Vizheh M., Qorbani M., Arzaghi S.M., Muhidin S., Javanmard Z., Esmaeili M. (2020). The mental health of healthcare workers in the COVID-19 pandemic: A systematic review. J. Diabetes Metab. Disord..

[25] Smallwood N., Karimi L., Bismark M., Putland M., Johnson D., Dharmage S.C., Barson E., Atkin N., Long C., Ng I. (2021). High levels of psychosocial distress among Australian frontline healthcare workers during the COVID-19 pandemic: A cross-sectional survey. Gen. Psychiatr..

[26] Dobson H., Malpas C.B., Burrell A.J.C., Gurvich C., Chen L., Kulkarni J., Winton-Brown T. (2020). Burnout and psychological distress amongst Australian healthcare workers during the COVID-19 pandemic. Australas. Psychiatry.

[27] Bell C., Williman J., Beaglehole B., Stanley J., Jenkins M., Gendall P., Rapsey C., Every-Palmer S. (2021). Challenges facing essential workers: A cross-sectional survey of the subjective mental health and well-being of New Zealand healthcare and ‘other’ essential workers during the COVID-19 lockdown. BMJ Open.

[28] Ministry of Health (2015). Health of the Health Workforce 2015: A report by Health Workforce New Zealand.

