# Supplementary material for: Mixed-Methods Survey of Healthcare Workers’ Experiences of Personal Protective Equipment during the COVID-19 Pandemic in Aotearoa/New Zealand
Source: Int J Environ Res Public Health. 2022 Feb 21;19(4):2474. doi: 10.3390/ijerph19042474 (PMC8874708; doi:10.3390/ijerph19042474)
Supplement: Supplementary file 1 [file ijerph-19-02474-s001.zip › ijerph-1573006-supplementary.pdf]

## SUPPLEMENTARY FILE S1

## ONLINE QUESTIONNAIRE OPEN QUESTIONS

- Have you been told not to wear PPE by your organisation for any other reason (other than stock levels)? What was the reason?
- If you purchased your own PPE, were you allowed to use it at work? If not, why not?
- Do you think frontline staff should have access to information regarding stock levels of PPE held by their organisation? Why/why not?
- If New Zealand faces further waves of COVID-19, are you confident that your workplace can provide all the necessary PPE you need? If not, why not?
- Has the COVID-19 pandemic made you more or less likely to remain in health as a profession?

## SUPPLEMENTARY TABLE S1

| Question domain | | Reported findings | Supporting data example |
|---|---|---|---|
| **Have you been told not to wear PPE by your organisation for any other reason (other than stock levels)? What was the reason?** | | It would create panic | *"Near the beginning, we were told not to wear PPE in some circumstances to avoid "alarming" the patients. I was also told on one occasion not to wear PPE while doing health checks at a quarantine facility because "it takes too long to change gloves and hand sanitise hands between patients and "you can't catch covid from someone's ear" (in relation to taking body temp)." – Nurse, female, 45-54 years, Māori* |
| | | It was unnecessary or staff were not at risk | *"Told not to wear PPE for any other cases (MRSA etc.) where we would normally wear gowns as they were 'no longer necessary' but we realised this was because there was a gown shortage, there was no evidence that we should not wear gowns for other infectious diseases that were not COVID-19." – Radiographer, female, 21-34 years, New Zealand European* |
| | | The cost of PPE was too high | *"Cost, our employer didn't want to pay for us to have masks etc." – Pharmacist, female, 21-34 years, New Zealand European* |
| **If you purchased your own PPE, were you allowed to use it at work? If not, why not?** | | It would create panic | *"We would frighten the patients if we wore masks and it wasn't necessary to wear a mask unless patient had symptoms or tested positive. Very scary times as doctors would bring their own masks but nursing staff told by* [organisation name omitted] *that no nurse should wear a mask." – Nurse, female, 55-64 years, New Zealand European* |
| | | It was deemed unnecessary | *"Was ridiculed and told it was unnecessary from Ministry of Health guidelines." – Health care assistant, female, 45-54 years, New Zealand European* |
| | | It was not approved by the workplace | *"It was not provided via supply lines that the organisation has approved and could be confident in." – Nurse practitioner, male, 35-44 years, New Zealand European* |
| | | There was a contamination risk | *"Work say, I've used it outside work and it could've contaminated* [it] *but disregarded my clothes that I wear outside." – Nurse, male, 35-44 years, Asian* |
| **Do you think frontline staff should have access to information regarding stock levels of PPE held by their** | Agree | It would help understand decision making | *"Gives staff confidence in how their organisation is managing the supply. Allows staff to understand why decisions are made and make their own judgement on what they should be doing / where the organisation is heading." – Dentist, male, 55-64 years, New Zealand European* |
| | | It would reassure healthcare workers | *"Communication and transparency reduces* [sic] *anxiety and misinformation spreading." – Dentist, female, 65+ years, New Zealand European* |
| | | It would help better prioritise resources | *"Would help orientate behaviour of staff at work to be more frugal with PPE use." – Early career doctor, female, 21-34 years, Asian* |
| | | It would promote trust | *"They promote transparency and it would build trust with staff." – Nurse, female, 35-44 years, Māori* |

| Question domain | | Reported findings | Supporting data example |
|---|---|---|---|
| **organisation? Why/why not?** | | It would make staff feel valued and part of a team | *"We are part of a team. If there are supply issues that affect individuals they need to be aware of this, so that they are able to participate in trouble shooting and problem solving. We are health care professionals and aware of nuances of individual work places, hence essential in deciding what areas need what protection." – Consultant doctor, female, 45-54 years, New Zealand European*<br>*"Team effort- everyone then feels empowered by their knowledge and understand the situation better." – Nurse, female, 45-54 years, Māori* |
| | | Staff have a right to the information | *"Ethical obligation of employer." – Consultant doctor, male, 35-44 years, New Zealand European*<br>*"We have a right to know these things. It's an insult to our morality and our intelligence to just keep this information locked away, and not allow us to use our clinical judgement." – Nurse, female, 21-34 years, Asian* |
| | | Staff could choose not to work if they felt unprotected | *"Staff should have the right to decline / defer their service within reason that (sic) they don't feel safe to properly do their job." – Occupational therapist, male, 35-44 years, Asian* |
| | Disagree | It would make people panic | *"It was panic central with pockets of very badly behaved individuals hoarding and or "stealing" PPE […] Showing stock levels would have caused worsening of this behaviour as stock levels were critically low." – Consultant doctor, male, 45-54 years, New Zealand European* |
| | | It would lead to behaviour change and policing of usage | *"I don't think it should be of any concern. If we knew and stock was running low we may have been tempted to skimp on keeping ourselves safe." – Dental hygienist, female, 45-54 years, New Zealand European* |
| | | It is not the responsibility of healthcare workers | *"Not their responsibility or worry. It is already an emotional period without worrying about something they have no control over. It is the organisation's responsibility to source and provide it." – Dentist, female, 55-64 years, New Zealand European*<br>*"Not my problem. If no PPE, then no patients treated." – Physiotherapist, female, 45-54 years, New Zealand European* |
| **If New Zealand faces further waves of COVID-19, are you confident that your workplace can provide all the necessary PPE you need? If not, why not?** | | Known global supply issues | *"World shortages of PPE and freighting services." – Dentist, female, 45-54 years, New Zealand European* |
| | | Churn rate is too high | *"Because we are already struggling for the appropriate PPE that we need on a daily basis." – Dental hygienist, female, 21-34 years, New Zealand European* |
| | | Have failed fit-tests | *"Currently, there is no N95 mask that fits me following discontinued supply of original* [brand name omitted] *masks. I have failed fit-tests with other manufacturer/versions." – Nurse, female, 45-54 years, New Zealand European*<br>*"Lots - around half of child health nurses at* [organisation name omitted] *are failing fit-testing for N95 and no alternative has been found yet." – Nurse, female, 35-44 years, New Zealand European* |

| Question domain | | Reported findings | Supporting data example |
|---|---|---|---|
| | | | *"Based on the first wave and based on the fact that my organisation has still not completed fit-testing for all staff and has still not given any indication of how those who failed the fit-testing by will be provided with appropriately fitting N95 masks or alternatives – Midwife, female, 35-44 years, New Zealand European* |
| | | Quality of available PPE is poor | *"The quality has also dropped. I order level 2 masks and they are not good quality. Different manufactures are putting out less quality products now." – Dental hygienist, female, 55-64 years, New Zealand European* |
| | | Lack of transparency | *"The first wave was enough to tell me so. Maybe if they'd communicated better, I'd probably feel different. They've done everyone, themselves a disservice by trying to keep it from us." – Nurse, female, 21-34 years, Māori* |
| | | Lack of trust in the organisation | *"I don't trust that the workers' wellbeing is a top priority with them." – Health care assistant, female, 45-54 years, Māori* |
| | | Feel dispensable | *"They* [organisation name omitted] *don't value staff. So that's why I buy my own now." – Nurse, female, 55-64 years, New Zealand European*<br>*"We are gagged by the DHB's media policies. No one gives a damn about us. When supplies get low, we'll just be expected to martyr ourselves." – Nurse, female, 21-34 years, Asian* |
| **Has the COVID-19 pandemic made you more or less likely to remain in health as a profession?** | More likely to remain, or no change to intent to remain | High job satisfaction | *"Healthcare is what I do. This is just another aspect of my work. Somewhat challenging times but maybe that's what makes it more rewarding." – Nurse, female, 55-64 years, New Zealand European*<br>*"I love what I do. What a time to start as a new graduate nurse in a world pandemic." – Nurse, female, 21-34 years, Māori* |
| | | Job security | *"Job security is extremely good where I work." – Administration, female, 21-34 years, Pacific Peoples* |
| | | Pandemics are 'part of the job' | *"In healthcare we face risk of infection daily many of which can be life-threatening. This is the job and it is important to take the correct precautions to protect yourself. COVID-19 does not change my desire to continue in this profession." – Nurse, female, 35-44 years, New Zealand European*<br>*"It's what we signed up to, it's literally our job, yes it has been tough but it's literally our job." – Nurse, female, 21-34 years, New Zealand European* |
| | | Have not felt at risk at work | *"I'm still relatively young and low risk." – Early career doctor, female, 21-34 years, Asian*<br>*"I feel I was away from the 'main front line' and had sufficient confidence in screening and testing to know that there were limited chance of exposure." – Dietitian, female, 35-44 years, New Zealand European* |
| | | No other options or near retirement | *"I would love to quit but have to pay the bills somehow. I don't feel valued at all by my* [organisation name omitted] *management - I'm a number to work where ever they want me." – Nurse, female, 35-44 years, New Zealand European* |

| Question domain | | Reported findings | Supporting data example |
|---|---|---|---|
| | | | *"I'm 62 and due to retire, too old to start again." – Occupational therapist, female, 55-64 years, New Zealand European* |
| | Less likely to remain, or no change to intent to leave | Profession is too risky | *"I don't feel safe, as simple as that. The hospitals main priority at all times seems to be budgetary concerns rather than the welfare of either its patients or staff." – Anaesthetic Technician, male, 45-54 years, Māori*<br>*"I didn't become a nurse to be put in a situation where my life is at risk." – Nurse, female, 55-64 years, New Zealand European* |
| | | High stress and risk of burnout | *"I am not prepared mentally for the stress of being a martyr." – Anaesthetic Technician, female, 21-34 years, Asian*<br>*"It did make me consider again if another industry might be better suited, I love health for the service I can provide but sometimes I think the effect on mental health and risks of burnout can be a bit too close to home and would affect being able to be of good service." – Audiologist, female, 21-34 years, Asian* |
| | | Feel undervalued and under-resourced | *"The Health System has always been a mess (for as long as I've worked in it). But this just highlights how little society gives a damn about health workers - everyone should have the right to come home from their jobs, unless you're a health worker, in which case this is what you signed up for. The failure of WorkSafe* [NZ's health and safety regulator] *to lay any charges or investigate how my colleagues got infected tells me all I need to know about what's expected of us in society. We say that life is valuable, that we should preserve life, but this doesn't apply for health workers." – Nurse, female, 21-34 years, Asian*<br>*"I've been nursing for 30+ years and have seen many changes. We are understaffed, under-resourced and yet are being constantly harangued to work harder, faster, smarter. I am in a senior position so although frontline, I am paid better than many and to be honest am loathe to give that up just yet. In saying that, should an equally attractively remunerated position become available I would be out of this DHB like a rat out of a hole." – Nurse, female, 55-64 years, New Zealand European* |
| | | Financially untenable [a] | *"Lack of government support for role* [of] *general practice. We stayed open at huge personal and financial cost." – General practitioner, female, 55-64 years, New Zealand European*<br>*"General dental practice is not economically viable at AL3* [NZ's COVID-19 alert level 3] [b] *without enormous fee increases for private patients. DHB contracts are not economic at any AL* [alert level] *but cannot be sustained without the private fee paying part of the practice subsidising them." – Dentist, male, 55-64 years, New Zealand European* |

DHB, District Health Board responsible for providing or funding the provision of health services in a given district in New Zealand; PPE, personal protective equipment. [a] Relating to those respondents in private practice; [b] Between March 2020 and December 2021, an alert level restrictions system was in place with four levels of escalating restrictions. Level 1 involved no restrictions, level 2 included limits on gatherings, and levels 3 and 4 were forms of lockdown with gathering and travel restrictions.
Quotes are ascribed to individual respondents, who are 'identified' by their demographic characteristics. The names from organisations were omitted in order to protect the anonymity of respondents.
